# Evaluating Different Supplements on the Growth Performance and Bioconversion Efficiency of Kitchen Waste by Black Soldier Fly Larvae

**DOI:** 10.3390/insects16010022

**Published:** 2024-12-29

**Authors:** Lifei Chen, Meng Xu, Rongsheng Shang, Yizhen Xin, Guiying Wang, Yifan Li, Zhuoya Wang, Xiangyu Wang, Haoyang Sun, Lusheng Li

**Affiliations:** College of Agriculture and Biology, Shandong Province Engineering Research Center of Black Soldier Fly Breeding and Organic Waste Conversion, Liaocheng University, Liaocheng 252000, China; 2210190104@stu.lcu.edu.cn (M.X.); 2110190115@stu.lcu.edu.cn (R.S.); 2210190117@stu.lcu.edu.cn (Y.X.); wangguiying@lcu.edu.cn (G.W.); l583613021@126.com (Y.L.); 2022404149@stu.lcu.edu.cn (Z.W.); 2022404144@stu.lcu.edu.cn (X.W.); 13210751109@163.com (H.S.)

**Keywords:** moisture content, maize stover, BSFL frass, body weight, feed conversion rate

## Abstract

Black soldier fly larvae (BSFL) convert kitchen waste into high-quality insect feed. However, the optimal amount of auxiliary materials needed to improve the physical and chemical properties of kitchen waste and enhance BSFL bioconversion efficiency remains unresolved. In this study, maize stover and BSFL frass were added to kitchen waste (in groups G2 and G3, respectively) to explore their effects on the growth performance and bioconversion efficiency of BSFL. The group with only kitchen waste, without the addition of maize stover or BSF frass, was used as the control group and labeled as G1. On the 5th day, the body length of the BSFL in the G2 group was significantly greater than that in G1 and G3 (*p* < 0.05). The dry matter weight loss rate in the G3 group was significantly lower compared to that of G1 and G2 (*p* < 0.05), and the feed conversion rate (FCR) of G1 was significantly lower than that of G2 and G3 (*p* < 0.01). In summary, adding maize stover and BSFL frass increased BSFL feed intake and improved body weight gain. However, these additives did not significantly enhance BSFL bioconversion efficiency. The organic matter in maize stover and BSFL frass was utilized by the BSFL, and the heavy metal levels in each group of BSFL did not exceed standard limits.

## Simple Summary

Black soldier fly larvae (BSFL) are useful for waste management and bioremediation owing to their fast growth rate and nutritious organic composition. However, traditional rearing methods do not consider optimizing the moisture content of kitchen waste feed to optimize their growth. This study explored the effects of adding maize stover and BSFL frass to kitchen waste on the growth performance and bioconversion efficiency of BSFL. Adding maize stover and BSFL frass adjusted the moisture content and air permeability of the kitchen waste, thereby increasing the BSFL feed intake and improving body weight gain. The use of maize stover and BSFL frass did not significantly enhance the bioconversion efficiency of the BSFL. This study provides further insights into managing the moisture content of kitchen waste feed for the rearing of BSFL to improve their growth.

## 1. Introduction

Black soldier fly larvae (BSFL) are an insect species known for their scavenging ability, characterized by a diverse diet, high bioconversion efficiency, rapid reproductive cycle, minimal space requirements for rearing, and the fact that adult flies do not require feeding [1,2,3]. The prepupae of BSFL are highly nutritious, boasting a well-balanced profile of essential amino acids and a high protein value [4]. These attributes make BSFL particularly valuable for waste management, as they can efficiently convert kitchen waste, livestock and poultry manure, spoiled fruits, and expired grains into larval biomass [5,6]. This biomass can then be repurposed as a protein-rich animal feed ingredient [7]. BSFL contain 40–48% crude protein and 25–40% ether extract [8], and their use as a protein source has shown promising results across various sectors, including livestock [9], poultry [10,11], and aquaculture [12,13,14].

Improving bioconversion efficiency is crucial for the sustainable production of BSFL. Single-source waste often presents challenges such as high crude fiber content [15], carbon/nitrogen (C/N) imbalances [16], and the presence of phenolic toxic substances [17], all of which can negatively impact bioconversion efficiency. Li et al. [18] achieved a significant improvement in BSFL bioconversion efficiency by using 30% bean curd residue as a substitute for kitchen waste, resulting in the highest bioconversion rate of 18.54% and the lowest feed conversion ratio (FCR) of 2.51. In a separate study, Isibika et al. [19] explored the effects of a mixture of banana peels, apple peels, and fish waste on the BSFL bioconversion rate. Their findings indicated that composting with pure and mixed banana and orange peels resulted in lower final larval weights and biomass conversion efficiency. However, when fish waste was combined with fruit waste, there was a notable increase in BSFL biomass conversion efficiency.

The moisture content of kitchen waste is another important factor that affects the growth and bioconversion efficiency of BSFL. The moisture content of kitchen waste influences the growth and development of BSFL [20], prolongs the prepupal period [21], and contributes to difficulty in the separation of the insect biomass from the residue since the residue can become too wet and too viscous for sieving [22]. Cheng et al. [23] demonstrated that the insect biomass can be effectively separated from the residue by sieving using a 2.36 mm sieve for both types of kitchen waste at 70% and 75% moisture content. However, studies have shown that sieving the residue is not feasible for kitchen waste with an 80% moisture content. Various pretreatment methods, such as mixing multiple types of organic waste in appropriate proportions, adjusting the C/N ratio, adding microorganisms to aid in conversion, and modifying the moisture content of the waste, can have different impacts on biomass conversion efficiency and economic value [19].

Maize stover is rich in organic carbon and is a common agricultural waste. It has a high lignocellulose content, leading to low biodegradability [24,25], which can affect the conversion efficiency of BSFL [26,27]. However, it can be used as a bulking material to increase the porosity of fine-textured organic waste [28] and improve the biomass conversion efficiency of BSFL. The bioconversion of maize stover using BSFL produced residual frass that met the Chinese organic fertilizer standard NY525-2012 [29].

BSFL frass is a by-product of BSFL rearing and is composed of spent feedstock, BSFL feces, and cuticles. After BSFL digestion and egestion, frass enhances the amount of water-extractable nitrogen in organic materials that can be used by vegetables for growth [30]. Fresh frass cannot be used directly as an organic fertilizer for vegetables and other plants because it contains harmful amounts of unconverted organic matter and plant toxins [31]. Replacing 10–20% of peat moss with fresh frass can promote the growth of lettuce, basil, and tomato. However, over 20% replacement will hinder the growth of these three vegetables [32]. Frass from kitchen waste has a moisture content of less than 35% and features good mobility and permeability. It also contains numerous digestive enzymes. Incorporating a certain proportion of frass along with pig manure into the feed for BSFL helps reduce the moisture content of the pig manure and breaks down macromolecular organic matter, which enhances the digestion and absorption efficiency of the BSFL [33]. This indicates that high-quality frass is a good auxiliary material for organic waste pretreatment.

Currently, the traditional rearing method for BSFL is to feed them kitchen waste directly without moisture adjustment, although the moisture content significantly varies depending on the type of kitchen waste. Therefore, it is necessary to add auxiliary materials to adjust the moisture content and improve the conversion efficiency. There are few reports on the use of frass and maize stover as adjuvants to adjust the water content and study the biomass conversion efficiency of BSFL. In this study, the effects of different proportions of maize stover, frass, and kitchen waste on the growth, bioconversion efficiency, prepupal nutrients, and heavy metal content of BSFL were determined. The main purpose was to find appropriate auxiliary materials to adjust the moisture content of kitchen waste and improve its conversion efficiency.

## 2. Materials and Methods

### 2.1. Materials

#### 2.1.1. Black Soldier Fly Larvae

Six-day-old BSFL were obtained from Shandong Woneng Agricultural Science and Technology Co., Ltd. (Liaocheng, China). The BSFL were preliminarily reared for 6 days on special feed composed of 15% corn flour, 70% bran, and 15% soybean meal. This feed has a metabolizable energy content of 11.5 MJ/kg and a moisture content of 80%. The feed was provided at a rate of 0.85 g per 100 larvae.

#### 2.1.2. Frass

Kitchen waste was collected from a canteen at Liaocheng University. After the kitchen waste is homogenized to a particle size of about 2–5 mm, 6-day-old BSFL were introduced into the mixture for their rearing. When the color of the kitchen waste changed and the texture became fluffy, the frass and larvae were separated using an air-blown vibrating sieve (Henan Fuyuan Machinery Manufacturing Co., Ltd., Zhengzhou, China). The frass was immediately dried in an oven at 65 °C for 24 h and then pulverized to a particle size of 2 mm with a grinding machine (RS-FS1401, Hefei Royalstar Small Appliances Co., Ltd., Hefei, China), left in the laboratory for 24 h, and then stored in a refrigerator at −20 °C until use.

#### 2.1.3. Maize Stover

Maize stover was provided by Shandong Woneng Agricultural Science and Technology Co., Ltd. (Liaocheng, China). It was crushed with a grinding machine (RS-FS1401, Hefei Royalstar Small Appliances Co., Ltd., Hefei, China) to a particle size of 2 mm, dried in an oven at 65 °C for 24 h, placed in the laboratory for 24 h, and stored in a refrigerator at −20 °C until use.

#### 2.1.4. Processing of the Materials

The control group (G1) only contained kitchen waste with 80.74% moisture. The maize stover test group (G2) and BSFL frass test group (G3) were prepared with 67.80% moisture. The relative content of each group was measured before use and is listed in Table 1.

### 2.2. Feeding Trial

The feeding trial was conducted at the Animal Feeding Laboratory of Liaocheng University. Each treatment was replicated three times. Two hundred 6-day-old BSFL with similar body sizes, along with 400 g of trial material, were added to each replicate in a round plastic container (diameter: 11.70 cm, height: 11.80 cm). The trial lasted for 7 days.

The research was conducted in a greenhouse at 30 ± 2 °C with 70% relative humidity conditions for 7 d. On days 1, 3, 5, and 7, the body weights and body sizes of 30 larvae were measured to determine BSFL growth and development. At the end of the trial, all larvae were manually separated from the residual material. The number of larvae was counted, and their fresh weight was recorded. The larvae were then washed in water, inactivated by heating at 110 °C for 10 min by oven (101-0S, Shaoxing Supo Instrument Co., Ltd., Shaoxing, China), and subsequently dried at 60 °C until they reached a constant weight. The remaining residue was weighed and dried at 105 °C until a stable mass was achieved.

### 2.3. Chemical Analysis

The total moisture content and dry mass of the fresh trial materials, BSFL, and residues were determined by drying at 105 °C, following the guidelines of the Chinese National Standard GB 5009.3-2010 [34]. Crude protein (CP) was measured using the Kjeldahl method, and a conversion factor of 6.25 was calculated using the method in GB/T 5009.5-2010 [34]. The ether extract (EE) of the larvae and trial materials was determined using Soxhlet extraction (GB/T 5009.6-2003) [34]. Crude ash (Ash) was determined following the National Standard GB 5009.4-2010. The concentrations of five heavy metals, including total arsenic (As), lead (Pb), mercury (Hg), cadmium (Cd), and chromium (Cr), were determined using an iCAP inductively coupled plasma mass spectrometer (ICP-MS) (ICP-MS, iCAPQ, Thermo Scientific, Waltham, MA, USA) in accordance with the corresponding Chinese National Standards: GB/T 13079-2006, GB/T 13080-2018, GB/T 13081-2006, GB/T 13082-1991, and GB/T 13088-2006 [35].

### 2.4. Processing Parameter Calculations

Based on previous studies, the BSFL biomass production parameters were survival rate, dry mass reduction, bioconversion rate, and feed conversion ratio (FCR).

Survival rate (%) = [number of larvae at the end of the trial/number of larvae at the beginning of the trial] × 100.

Dry mass reduction (%) = [(mass of material at the beginning of the trial (g) − mass of residue at the end of the trial (g))/mass of material at the beginning of the trial (g)] × 100.

Bioconversion rate (%) = [total larval biomass (g)/trial material added (g)] × 100.

FCR = mass of the ingested trial material (g)/mass gained (g).

### 2.5. Statistical Analysis

All the trial data were recorded and organized in Excel 2019, and mean values and standard deviations were calculated. Meanwhile, the data distribution was preliminarily verified for normality and homoscedasticity. Statistical analyses were conducted using SPSS (version 17.0; SPSS Inc., Chicago, IL, USA, 2008). A one-way analysis of variance (ANOVA) was used to analyze the results of all experiments. Duncan’s method was used to test the difference between the mean values of each parameter, with a significance level of *p* < 0.05. The data were expressed as the mean ± S.E.

## 3. Results

### 3.1. Growth Performance of BSFL Under Different Treatment Conditions

The body weights of the BSFL significantly increased in G2 (20% maize stover) and G3 (20% BSFL frass) on days 3, 5, and 7 compared to G1 (*p* < 0.05, *p* < 0.01, *p* < 0.05, respectively) (Table 2). No significant difference was observed between groups G2 and G3 (*p* > 0.05). In terms of body length, the body length of the BSFL in G2 was significantly higher than that in G1 and G3 (*p* < 0.05), but only on day 5.

### 3.2. Dry Mass Reduction, Bioconversion Rate, Survival Rate, and FCR of BSFL Under Different Treatment Conditions

The dry mass reduction in G3 was significantly lower than that in G1 and G2 (*p* < 0.05) (Table 3). The bioconversion rates in G2 and G3 were significantly lower than that in the control G1 (*p* < 0.05), decreasing by 10.68% and 11.16%, respectively. The survival rate of the BSFL in each group was not significantly different (*p* > 0.05). The BSFL FCR of the G2 and G3 groups was significantly increased by 17.25% and 20.89% compared with that of the control group, respectively (*p* < 0.01).

### 3.3. Nutrients and Heavy Metal Content of BSFL Under Different Treatment Conditions

As shown in Table 4, there was no significant difference in crude ash content among the G1, G2, and G3 groups (*p* > 0.05). The ether extract content in G1 was significantly higher than that in G3 (*p* < 0.05) and no significant difference was observed between groups G2 and G3 (*p* > 0.05). In terms of crude protein content, there was no significant difference between G2 and G1 (*p* > 0.05), nor between G3 and G1 (*p* > 0.05), but G2 was significantly higher than G3 (*p* < 0.05). There was no significant difference in heavy metal content among the groups (*p* > 0.05), but the data for G3 tended to be higher than those for G1 and G2.

## 4. Discussion

In this study, maize stover and BSFL frass were added to kitchen waste to adjust its moisture content to 67.80%, and BSFL were then fed on it for seven days. Our results indicated that the addition of maize stover and BSFL frass to kitchen waste significantly improved BSFL body weight on days 3, 5, and 7, as well as increased the FCR of the BSFL. The enhanced growth performance of the BSFL with the addition of maize stover and BSFL frass was likely due to increased food intake by the larvae. This improvement in growth was independent of the conversion efficiency of kitchen waste. Most studies suggest that adding auxiliary materials to kitchen waste can improve BSFL growth and development [36,37,38]. For instance, adding a certain proportion of bean curd residue to kitchen waste significantly enhances BSFL growth, with a 30% substitution rate achieving the lowest FCR of 2.51 [18]. The results of the present study were consistent with the previous findings regarding larval growth [18], but the outcomes differed for the FCR. This discrepancy is likely due to differences in the types of auxiliary materials used, the moisture content of the mixture, and the preparation process.

The moisture content of the substrate directly impacts the water requirements, consumption, and absorption by insects, which in turn influences their metabolic performance, growth, and development [39]. Black soldier fly larvae are scavenger insects that prefer moist and soft feed due to their specialized mouthparts [40]. Maintaining an appropriate substrate moisture level facilitates easier chewing and swallowing for the larvae, which in turn enhances their growth and survival [41,42]. If the substrate moisture content is too low, it causes water deficiency stress in the BSFL, hindering normal growth and development [43]. High moisture content dilutes the nutrient concentration per unit volume of feed. Therefore, the larvae reserve sufficient nutrients for development and reproduction by increasing their feed intake and prolonging the prepupal period [44]. A 60–80% moisture content of kitchen waste is optimal for larval growth, development, and feed conversion efficiency [23,45]. The total prepupa yield of BSFL increases linearly with higher moisture content between 40–80%, and the prepupa have the shortest duration at level of 70% [20]. Black soldier fly larvae have the greatest weight gain at a moisture content range of 40–60% when using chicken manure [46]. In this study, the moisture content of the pure kitchen waste group was 80.74%, while the moisture content for the maize stover powder and BSFL frass groups was 67.80%. The final experimental results indicated that the body weight of BSFL in the G2 and G3 groups increased by 65% compared to the G1 group. This is likely due to the kitchen waste with a moisture content of 67.80% significantly enhancing BSFL feed intake and accelerating their growth rate.

In recent years, several studies have used maize stover as a substrate in feeding trials with BSFL. However, maize stover is used as a preferred substrate in feeding trials, wherein lignin, cellulose, hemicellulose, and other substances can be degraded to promote BSFL feeding intake [25,29,47,48]. Adding a certain proportion of stover enhances the porosity of kitchen waste, which may facilitate BSFL respiration and movement in feeding substrates, thus improving the BSFL feeding intake and conversion rate [28]. In this study, maize stover was directly added to kitchen waste without any fermentation treatment. The body weight of the BSFL in this group was significantly higher than that of the pure kitchen waste group, which may be related to the increase in material porosity. However, the ratio of feed to insects was higher than that in the pure kitchen waste group, indicating that the nutritional components of the maize stover could not be used by the BSFL.

The treatment of organic waste using BSFL can be considered a novel approach. Black soldier fly larvae can effectively decompose various types of organic wastes and convert them into high-value biomass. Improving the BSFL survival rate and bioconversion efficiency are important factors in the BSF industry [22,49]. Feed consumption by BSFL is a result of the combined action of microorganisms and larvae [50]. Microbial communities are active when the moisture content of the feed for BSFL is between 40–60% [51]. These communities compete with BSFL for feed, reducing the available nutrient content, which is not conducive for the accumulation of nutrients in larvae. The ideal moisture content of the feed for BSFL was in the range of 60–80% [23,45]. The level of available oxygen may be reduced owing to the high moisture content, which hinders the metabolism of microorganisms. However, moisture content can influence BSFL growth and nutrient accumulation by affecting the feed temperature. Microorganisms decompose the feed substrate and produce heat, which causes the compost temperature to rapidly rise to above 35 °C, exceeding the optimal growth temperature of larvae (27–35 °C), causing high temperature stress in the larvae [8,52].

In this study, the BSFL survival rate was not significantly different among the treatments; however, the BSFL biomass conversion rate was significantly reduced when maize stover and BSFL frass were added to kitchen waste. The dry matter weight loss rate of the frass BSFL group was also significantly lower than that of the pure kitchen waste and maize stover groups. Under our experimental conditions, the differences in moisture content did not affect the survival rate or bioconversion efficiency of BSFL. The unfermented maize stover and dried BSFL frass might influence the original active components and have a lower nutritional value than kitchen waste.

Black soldier fly larvae meal contains high-quality crude protein [53,54] and can replace traditional protein feed ingredients such as fish meal and soybean meal [55,56]. The growth performance and antioxidant capacity of laying hens could be significantly improved when an appropriate amount of defatted BSFL meal was added to optimize the formula in an equal energy and protein diet [57]. The inclusion of 3% BSFL does not affect the growth performance of Chinese soft-shelled turtles [14]. In the present study, the crude protein content of the BSFL frass group was significantly lower than that of the maize stover group, and the ether extract contents were 12.16% and 10.19% lower than those of the pure kitchen waste and maize stover groups, respectively. There was no difference between the BSFL frass and maize stover groups, which is similar to findings in previous studies [33].

The deposition of protein and fat in BSFL is closely related to feed quality [19,58] and the growth environment [52]. Low moisture causes stress in BSFL, which preferentially use carbohydrates and fats in the body to provide more energy to resist adverse external effects [20]. Under high-moisture conditions, larvae can increase feed intake and convert excess carbohydrates into lipids, which have higher energy values than proteins and carbohydrates; thus, the total energy and EE content increase linearly. In this study, the protein and fat contents of BSFL frass were significantly lower than those of the other two groups, indicating that BSFL frass could not be utilized by BSFL, despite containing a certain amount of organic matter. There was no difference between the maize stover and pure kitchen waste groups, indicating that the organic matter in the maize stover was partially utilized by the BSFL, and that the specific utilization route might be direct utilization or microbial fermentation during feeding [48].

The composition of kitchen waste is complex. In addition to leftover food and its by-products, kitchen waste often contains plastic bags, paper towels, lunch boxes, chopsticks, metals, and detergents. Therefore, there is a potential risk of heavy metals being present in kitchen waste [59]. The collection of kitchen waste and its different treatment methods may lead to varying degrees of heavy metals that may accumulate in BSFL biomass and enter the food chain, limiting their use as animal feed [60]. Shi et al. collected and analyzed 60 heavy metals in BSFL fed with different kitchen wastes and found that total arsenic, lead, mercury, chromium, and cadmium were all detected but did not exceed the limit [35]. The heavy metal content in BSFL frass negatively correlates with that in BSFL biomass [61]. Although there were no significant differences among the treatments in this study, the total arsenic, lead, mercury, chromium, and cadmium in the BSFL frass group increased, and there was a certain degree of risk from heavy metals in the BSFL frass. Therefore, it is necessary to pay close attention to BSFL frass when returning the feed.

## 5. Conclusions

In this study, in order to reduce the moisture of the rearing substrate, the impact of adding maize stover and BSFL frass to the kitchen waste was investigated. The results showed that the addition of maize stover and BSFL frass to kitchen waste reduced the moisture content of the waste and significantly increased the body weight of BSFL by 9.95%, and 7.96%, respectively. At the same time, the BSFL FCR of the G2 and G3 groups was significantly increased by 17.25% and 20.89% compared with the control group, respectively. In addition, there was no significant difference in the heavy metal content between the control group and the experimental group. It is inferred that the addition of maize stover and BSFL frass can adjust the moisture content and air permeability of kitchen waste, thereby increasing the BSFL intake and improving weight gain. This study provides further insights into managing the moisture content of kitchen waste feed to enhance BSFL growth.

## Figures and Tables

**Table 1:** Nutritional constituents of the trial material under wet weight conditions among all treatments.

Treatment	Proportion of KW (%)	Moisture Content (%)	CP (%)	EE (%)	As (ppm)	Pb (ppm)	Hg (ppb)	Cd (ppm)	Cr (ppm)
G1	100	80.74	30.12	19.73	0.11	0.09	2.16	0.06	0.16
G2	80	67.80	24.86	15.81	0.09	0.07	1.75	0.05	0.13
G3	80	67.80	25.44	16.07	0.12	0.09	2.31	0.06	0.20

G1: pure kitchen waste; G2: 20% maize stover; G3: 20% black soldier fly (BSF) frass; KW, kitchen waste; CP, crude protein; EE, ether extract; As, arsenic; Pb, lead; Hg, mercury; Cd, cadmium; and Cr, chromium.

**Table 2:** The effects of maize stover and frass on BSFL growth.

Treatment	Body Weight (mg)					Body Length (mm)			
	1 d	3 d	5 d	7 d	Total Weight Gain	1 d	3 d	5 d	7 d
G1	1.82 ± 0.39	37.71 ± 1.49 ^a^	106.35 ± 1.52 ^A^	146.50 ± 6.16 ^a^	144.68 ± 3.59 ^a^	3.26 ± 0.01	6.46 ± 0.14	10.14 ± 0.15 ^a^	15.87 ± 0.38
G2	1.79 ± 0.73	43.63 ± 2.06 ^b^	121.94 ± 1.88 ^B^	160.88 ± 1.81 ^b^	159.08 ± 1.77 ^b^	3.27 ± 0.04	6.53 ± 0.30	12.13 ± 0.58 ^b^	16.12 ± 0.18
G3	1.78 ± 0.17	45.14 ± 1.19 ^b^	125.30 ± 2.08 ^B^	157.97 ± 1.32 ^b^	156.19 ± 1.30 ^b^	3.18 ± 0.09	6.81 ± 0.36	10.49 ± 0.50 ^a^	15.98 ± 0.13

^a,b^ Values within a column with no common letters significantly differ (*p* < 0.05). ^A,B^ Values within a column with no common letters significantly differ (*p* < 0.01). Each value represents the mean of three replicates.

**Table 3:** Survival rate of BSFL. Dry matter weight loss, bioconversion rate, and FCR of BSFL converting the trial material.

Treatment	Dry Matter Weight Loss (%)	Bioconversion Rate (%)	Survival Rate (%)	FCR
G1	72.73 ± 0.36 ^a^	14.79 ± 0.28 ^a^	99.17 ± 0.60	6.32 ± 0.04 ^A^
G2	72.08 ± 0.17 ^a^	13.21 ± 0.69 ^b^	99.33 ± 0.44	7.41 ± 0.19 ^B^
G3	70.48 ± 0.90 ^b^	13.14 ± 0.33 ^b^	99.33 ± 0.17	7.64 ± 0.23 ^B^

^a,b^ Values within a column with different letters differ significantly (*p* < 0.05). ^A,B^ Values within a column with different letters differ significantly (*p* < 0.01). FCR, feed conversion ratio. Each value represents the mean of three replicates.

**Table 4:** The nutritional composition and heavy metal content of BSFL.

Treatment	Ash (%)	EE (%)	CP (%)	As (ppm)	Pb (ppm)	Hg (ppb)	Cd (ppm)	Cr (ppm)
G1	9.86 ± 1.11	28.50 ± 0.74 ^a^	48.65 ± 1.18 ^ab^	0.40 ± 0.01	0.28 ± 0.02	7.57 ± 0.55	0.14 ± 0.02	0.36 ± 0.02
G2	10.94 ± 1.55	28.00 ± 1.70 ^ab^	49.19 ± 1.16 ^a^	0.39 ± 0.02	0.27 ± 0.02	7.07 ± 0.18	0.14 ± 0.01	0.34 ± 0.02
G3	12.02 ± 0.79	25.41 ± 0.80 ^b^	48.20 ± 1.48 ^b^	0.43 ± 0.02	0.33 ± 0.03	7.70 ± 0.49	0.18 ± 0.01	0.41 ± 0.01

^a,b^ Values within a column with no common letters differ significantly (*p* < 0.05). Ash, crude ash; CP, crude protein; EE, ether extract; As, arsenic; Pb, lead; Hg, mercury; Cd, cadmium; and Cr, chromium. Each value represents the mean of three replicates.

## Data Availability

The data presented in this study are available upon request from the corresponding author. The data are not publicly available because of companies’ policies.
